# Prone position improves lung ventilation–perfusion matching in non-intubated COVID-19 patients: a prospective physiologic study

**DOI:** 10.1186/s13054-022-04069-y

**Published:** 2022-06-29

**Authors:** Ling Liu, Jianfeng Xie, Changsong Wang, Zhanqi Zhao, Yang Chong, Xueyan Yuan, Haibo Qiu, Mingyan Zhao, Yi Yang, Arthur S. Slutsky

**Affiliations:** 1 Jiangsu Provincial Key Laboratory of Critical Care Medicine, Department of Critical Care Medicine, Zhongda Hospital, School of Medicine, Southeast University, 87 Dingjiaqiao Road, Nanjing, 210009 People’s Republic of China; 2 Department of Critical Care Medicine, The First Affiliated Hospital of Harbin Medical University, Heilongjiang Province, Harbin, People’s Republic of China; 3 Institute of Technical Medicine, Furtwangen University, Villingen-Schwenningen, Germany; 4 Department of Biomedical Engineering, Fourth Military Medical University, Xi’an, People’s Republic of China; 5 Interdepartmental Division of Critical Care Medicine, University of Toronto, Toronto, Canada; 6 Division of Respirology and Critical Care Medicine, Unity Health Toronto, Toronto, Canada; 7 Keenan Research Center, Li Ka Shing Knowledge Institute, St. Michael’s Hospital, Toronto, Canada

## To the Editor

Prone positioning may recruit gas exchange-efficient regions for typical acute respiratory distress syndrome (ARDS) and improve oxygenation. It is also a mainstay of treatment in COVID-19-related ARDS (C-ARDS) and reduces the need for intubation without any signal of harm [1]. In the early phase of COVID-19, hypoxemia may be caused by impaired regulation of pulmonary perfusion [2]. Whether awake prone positioning can improve ventilation/perfusion (V/Q) matching through redistribution of pulmonary perfusion has not been demonstrated. In this study, we assessed the effect of prone position on V/Q matching using electrical impedance tomography (EIT) in non-intubated COVID-19 patients.

Inclusion criteria were: age > 18 and ≤ 75 years, admitted to ICU with confirmed COVID-19-related pneumonia, receiving supplemental oxygen (standard oxygen therapy or high-flow nasal cannula (HFNC)) for < 24 h. Exclusion criteria are given in Additional file 1.

Demographic, anthropometric and baseline data (arterial blood gases and ventilation parameters) were collected while the patient was supine (time-point SP1). EIT measurement was performed with PulmoVista 500 (Dräger Medical GmbH, Lübeck, Germany). The patients received instructions as to how to perform an end expiratory occlusion lasting at least 10 s. About one second after the start of the occlusion, a bolus of 10 mL 10% NaCl solution was injected via the central venous catheter manually. Subsequently, each patient was helped to change position from supine to prone; this took several minutes. After approximately 1 h, data were collected during an end expiratory occlusion and NaCl injection (time-point PP). The patient was then encouraged to maintain the prone position for > 3 h (ranged from 3 to 5.8 h without interruption) before being returned to the supine position. Clinical data collection, end expiratory occlusion and injection were repeated 1 h after resupination (time-point SP2).

From offline analyses of EIT data obtained 5 min before and during saline bolus injection, we calculated V/Q matching as described previously [3]. V/Q matching was quantified as percentage of pixels classified as ventilated divided by the number of pixels classified as perfused. Pixels were denoted as dead space if they were identified as ventilated but not perfused. Pixels were denoted as shunt if they were identified as perfused but not ventilated. To quantify the relative contribution of the dead space versus shunt fraction to V/Q mismatch, we calculated the dead space-to--shunt ratio.

Fourteen patients with bilateral pneumonia were enrolled (Additional file 1: Table S1).

**Fig. 1:**
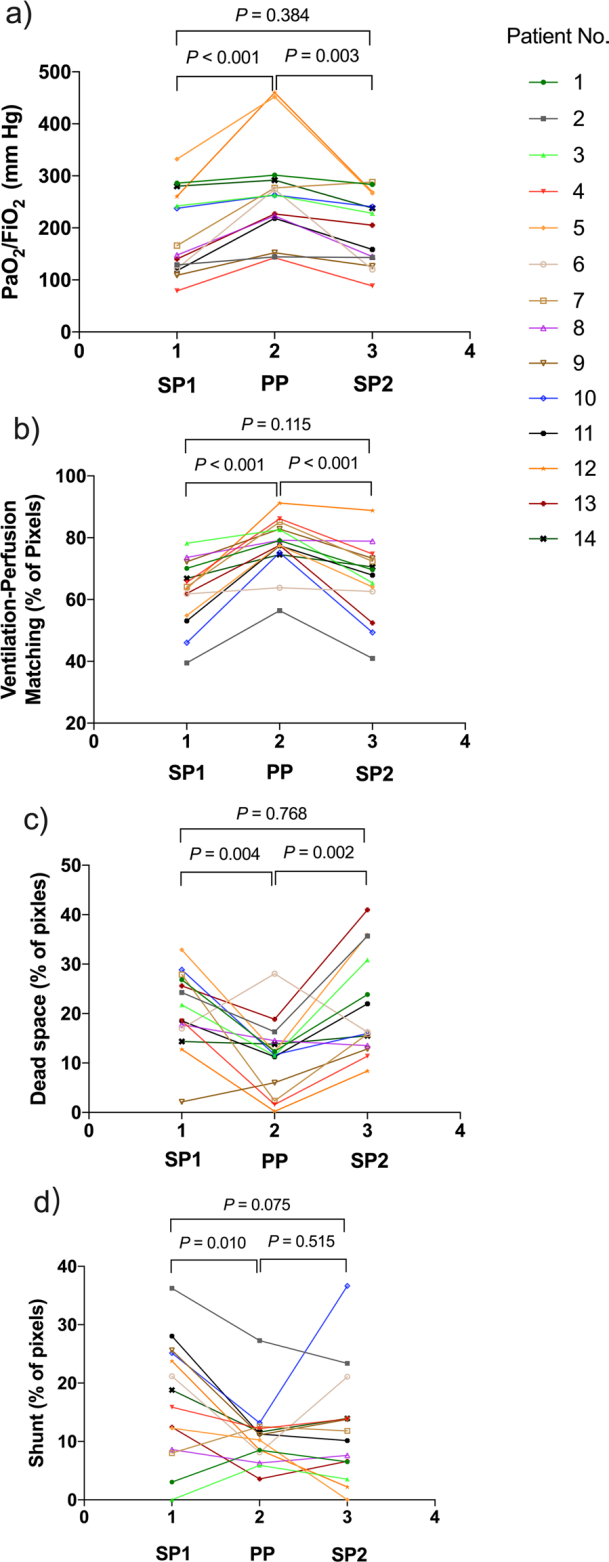
Per-patient trajectory of PaO_2_/FiO_2_ (**a**), ventilation–perfusion matching (**b**), dead space (**c**) and shunt (**d**) at the three study time points, SP1, PP and SP2. Each line is the trajectory of one patient. PaO_2_, partial pressure of oxygen; FiO_2_, fractional concentration of oxygen in inspired air; SP1, baseline supine position; PP, 1 h after prone positioning; SP2, 1 h after resuming supine position

V/Q matching, dead space fraction and oxygenation improved at PP, but were not maintained 1 h after resupination (Fig. 1). Prone positioning improved V/Q matching and PaO_2_/FiO_2_ in all the patients. Eight out of 14 patients had increased PaO_2_/FiO_2_ from SP1 to SP2. The dead space-to-shunt ratio was 1.9 ± 2.3, 1.4 ± 1.4 and 2.6 ± 2.5 at SP1, PP and SP2 (*P* = 0.392). Prone positioning decreased dead space fraction in 12 patients, decreased shunt fraction in 11 and decreased both shunt and dead space in 9. Dorsal ventilation and dorsal perfusion increased slightly during prone, and the respiratory rate was comparable (Additional file 1: Table S2).

These spontaneously breathing patients with COVID-19 pneumonia were characterized by reduced V/Q matching and a higher percentage of dead space lung units compared to shunt lung units. Prone positioning substantially improved overall V/Q matching mainly through decreased dead space, although this effect was lost after reverting to the supine position.

Hypoxemia in COVID-19 patients is primarily due to derangements in gas exchange caused by regional mismatch between ventilation and perfusion. Our data provide further evidence of V/Q mismatching, mainly due to nonperfused but ventilated units. The underlying biological mechanism might be microthrombi formation reflected by elevated d-dimers and loss of regulation of perfusion possibly by loss of hypoxic vasoconstriction, which may represent a key pathophysiological trait of C-ARDS.

The improvement in V/Q matching in the prone position was not surprising. Gravity is one of the variables responsible for V/Q matching, in concert with lung structure and fractal geometry, gas distribution and regulation of lung vascular tone [4]. The decrease in percentage of dead space lung units and slightly declining percentage of shunt lung units in our study during prone positioning might be related to several mechanisms including more uniform distribution of perfusion and ventilation in the prone position. Greater vascular conductance and greater nitric oxide expression in dorsal than in ventral lung vasculature might contribute to perfusion redistribution from supine to prone position [5]. There are other mechanisms impacting gas exchange in the prone position: pulmonary compression by the heart is decreased, changes in diaphragmatic function, and more uniform distribution of ventilation due to changes in elastance of the lung and chest wall.

This study has strengths in that it is the first to address the mechanisms leading to changes in gas exchange in the supine versus prone in spontaneously breathing patients not receiving CPAP. However, the major weakness is the small sample size and lack of a non-C-ARDS comparator group.

## Supplementary Information

**Additional file 1**. Supplemental data including exclusion criteria, EIT measurement, statistical analyses, main characteristics of the study population, as well as Respiratory physiology, blood gas and ventilation homogeneity parameters.

## Data Availability

The datasets used and/or analyzed during the current study are available from the corresponding author on reasonable request.

## Abbreviations

ARDS: Acute respiratory distress syndrome
C-ARDS: COVID-19-related ARDS
V/Q: Ventilation/perfusion matching
EIT: Electrical impedance tomography
HFNC: High-flow nasal cannula
SP1: Baseline supine position
PP: 1 H after prone positioning
SP2: 1 H after resuming supine position

## References

[1] Ehrmann S, Li J, Ibarra-Estrada M, Perez Y, Pavlov I, McNicholas B (2021). Awake prone positioning for COVID-19 acute hypoxaemic respiratory failure: a randomised, controlled, multinational, open-label meta-trial. Lancet Respir Med.

[2] Gattinoni L, Chiumello D, Caironi P, Busana M, Romitti F, Brazzi L (2020). COVID-19 pneumonia: different respiratory treatments for different phenotypes?. Intensive Care Med.

[3] He H, Chi Y, Long Y, Yuan S, Zhang R, Frerichs I (2020). Bedside evaluation of pulmonary embolism by saline contrast electrical impedance tomography method: a prospective observational study. Am J Respir Crit Care Med.

[4] Lindahl SGE (2020). Using the prone position could help to combat the development of fast hypoxia in some patients with COVID-19. Acta Paediatr.

[5] Nyrén S, Radell P, Lindahl SGE, Mure M, Petersson J, Larsson SA (2010). Lung ventilation and perfusion in prone and supine postures with reference to anesthetized and mechanically ventilated healthy volunteers. Anesthesiology.

