# Supplementary material for: Prone position improves lung ventilation–perfusion matching in non-intubated COVID-19 patients: a prospective physiologic study
Source: Crit Care. 2022 Jun 29;26:193. doi: 10.1186/s13054-022-04069-y (PMC9241304; doi:10.1186/s13054-022-04069-y)
Supplement: Supplementary file 1 — Additional file 1. Supplemental data including exclusion criteria, EIT measurement, statistical analyses, main characteristics of the study population, as well as Respiratory physiology, blood gas and ventilation homogeneity parameters. [file 13054_2022_4069_MOESM1_ESM.docx]

# Prone position improves lung ventilation-perfusion matching in non-intubated COVID-19 patients

**Online supplement**

**Methods**

Exclusion criteria were: altered mental status or uncooperative patient, New York Heart Association class III or greater, history of severe chronic obstructive pulmonary disease, contraindications to EIT (e.g., pacemaker or chest surgical wound dressing), patient with impending intubation (based on clinical judgment).

Once enrolled, an EIT belt containing 16 electrodes was placed around the patient’s chest at the level of the fifth intercostal space and connected to an EIT monitor (PulmoVista 500; Dräger Medical GmbH, Lübeck, Germany).

The same oxygen delivery (device and FiO_2_) was maintained and no sedation was given during the study.

All statistical analyses were carried out using SAS9.4 (SAS Institute Inc., Cary, NC, USA). Variables were compared using repeated measures analysis of variance, followed by a Bonferroni post-hoc test for comparison between different timepoints in Figure 1 and Table E2. P < 0.05 defined statistical significance.

Table E1 Main characteristics of the study population

| Patient | Sex | Age, (years) | Height (cm) | Real body weight (kg) | Comorbidities | Duration of symptom onset to ICU admission, (days) | Range of pneumonia | Type of supplemental oxygen | Fi0_2_ (%) | HFNC flow rate (L/min) |
| --- | --- | --- | --- | --- | --- | --- | --- | --- | --- | --- |
| 1 | Male | 55 | 167 | 61 | None | 3 | bilateral | Nasal cannula oxygen | 29 | - |
| 2 | Male | 68 | 169 | 67.5 | Hypertension, Diabetes | 3 | bilateral | HFNC | 40 | 40 |
| 3 | Female | 66 | 160 | 55 | Hypertension, Coronary heart disease | 7 | bilateral | Nasal cannula oxygen | 29 | - |
| 4 | Male | 68 | 170 | 66 | Hypertension | 11 | bilateral | HFNC | 70 | 50 |
| 5 | Female | 52 | 154 | 63 | None | 7 | bilateral | Nasal cannula oxygen | 29 | - |
| 6 | Male | 59 | 172 | 68 | Hypertension, Coronary heart disease | 11 | bilateral | HFNC | 50 | 50 |
| 7 | Male | 57 | 170 | 80 | None | 8 | bilateral | HFNC | 60 | 50 |
| 8 | Female | 68 | 170 | 60 | None | 1 | bilateral | Nasal cannula oxygen | 33 | - |
| 9 | Female | 71 | 165 | 57.5 | None | 7 | bilateral | HFNC | 50 | 40 |
| 10 | Female | 65 | 165 | 65 | Hypertension | 6 | bilateral | HFNC | 30 | 50 |
| 11 | Female | 73 | 155 | 65 | None | 8 | bilateral | HFNC | 45 | 50 |
| 12 | Male | 75 | 173 | 68 | None | 5 | bilateral | HFNC | 28 | 40 |
| 13 | Male | 72 | 170 | 72 | None | 12 | bilateral | HFNC | 30 | 50 |
| 14 | Male | 75 | 166 | 65 | Hypertension | 10 | bilateral | HFNC | 30 | 50 |
| mean (SD) | 8Male/ 6 Female | 66±8 | 166±6 | 65±6 | - | 8±3 | bilateral | 4 Nasal cannula oxygen / 10 HFNC | 40±14 | 47±5 |

Data are mean (SD), Fi0_2_ fraction of inspired oxygen

HFNC = High-flow nasal cannula oxygen

Table E2. Respiratory physiology, blood gas and ventilation homogeneity measured by Electrical Impedance Tomography at the three study time points.

|  | SP1 | PP | SP2 | p value |
| --- | --- | --- | --- | --- |
| Heart rate, beats per min | 82 ± 13 | 82 ± 16 | 78 ± 15 | 0.797 |
| Respiratory rate, breaths per min | 22 ± 5 | 23 ± 4 | 25 ± 7 | 0.301 |
| Mean arterial pressure, mm Hg | 89 ± 10 | 90 ± 8 | 91 ± 10 | 0.863 |
| Arterial blood gas |  |  |  |  |
| pH | 7.48 ± 0.04 | 7.47 ± 0.04 | 7.47 ± 0.03 | 0.499 |
| PaO_2_/FiO_2_, mm Hg | 189 ± 81 | 263 ± 97 ^* †^ | 200 ± 68 | 0.048 |
| PaCO_2_, mm Hg | 34 ± 4 | 36 ± 4 | 36 ± 4 | 0.311 |
| Lactate, mmol/L | 1.4 ± 0.5 | 1.2 ± 0.5 | 1.2 ± 0.4 | 0.385 |
| Ventilation-perfusion matching (% of pixels) | 62 ± 11 | 78 ± 9 ^* †^ | 67 ± 12 | 0.001 |
| Dead space fraction (% of pixels) | 21 ± 8 | 11 ± 7 ^*†^ | 21 ± 10 | 0.007 |
| Shunt fraction (% of pixels) | 17 ± 10 | 11 ± 6 | 12 ± 10 | 0.149 |
| Dead space to shunt ratio | 1.9 ± 2.3 | 1.4 ± 1.4 | 2.6 ± 2.5 | 0.392 |
| Dorsal ventilation, (% of pixels) | 56 ± 12 | 58 ± 12 | 52 ± 12 | 0.387 |
| Dorsal perfusion, (% of pixels) | 54 ± 10 | 55 ± 10 | 52 ± 13 | 0.676 |

SP1 = baseline supine position, PP= 1 hour after prone positioning. SP2 = 1 hour after resuming supine position, FiO_2_ = fractional concentration of oxygen in inspired air. PaCO_2_ = arterial partial pressure of carbon dioxide. PaO_2_ = arterial partial pressure of oxygen, SpO_2_ = peripheral oxygen saturation of haemoglobin,

^*^ p < 0.05 for diﬀerence between SP1 vs. PP

^†^ p *<* 0.05 for diﬀerence between PP vs. SP2

^‡^ p < 0.05 for diﬀerence between SP1 vs. SP2
